# Measures of Interjoint Coordination Post-stroke Across Different Upper Limb Movement Tasks

**DOI:** 10.3389/fbioe.2020.620805

**Published:** 2021-01-28

**Authors:** Anne Schwarz, Janne M. Veerbeek, Jeremia P. O. Held, Jaap H. Buurke, Andreas R. Luft

**Affiliations:** ^1^Vascular Neurology and Neurorehabilitation, Department of Neurology, University Hospital Zurich, University of Zurich, Zurich, Switzerland; ^2^Biomedical Signals and Systems (BSS), University of Twente, Enschede, Netherlands; ^3^Roessingh Research and Development B.V., Enschede, Netherlands; ^4^Cereneo, Center for Neurology and Rehabilitation, Vitznau, Switzerland

**Keywords:** upper extremity, stroke, biomechanical phenomena, kinematics, interjoint coordination

## Abstract

**Background:** Deficits in interjoint coordination, such as the inability to move out of synergy, are frequent symptoms in stroke subjects with upper limb impairments that hinder them from regaining normal motor function. Kinematic measurements allow a fine-grained assessment of movement pathologies, thereby complementing clinical scales, like the Fugl–Meyer Motor Assessment of the Upper Extremity (FMMA-UE). The study goal was to investigate the effects of the performed task, the tested arm, the dominant affected hand, upper limb function, and age on spatiotemporal parameters of the elbow, shoulder, and trunk. The construct validity of the metrics was examined by relating them with each other, the FMMA-UE, and its arm section.

**Methods:** This is a cross-sectional observational study including chronic stroke patients with mild to moderate upper limb motor impairment. Kinematic measurements were taken using a wearable sensor suit while performing four movements with both upper limbs: (1) isolated shoulder flexion, (2) pointing, (3) reach-to-grasp a glass, and (4) key insertion. The kinematic parameters included the joint ranges of shoulder abduction/adduction, shoulder flexion/extension, and elbow flexion/extension; trunk displacement; shoulder–elbow correlation coefficient; median slope; and curve efficiency. The effects of the task and tested arm on the metrics were investigated using a mixed-model analysis. The validity of metrics compared to clinically measured interjoint coordination (FMMA-UE) was done by correlation analysis.

**Results:** Twenty-six subjects were included in the analysis. The movement task and tested arm showed significant effects (*p* < 0.05) on all kinematic parameters. Hand dominance resulted in significant effects on shoulder flexion/extension and curve efficiency. The level of upper limb function showed influences on curve efficiency and the factor age on median slope. Relations with the FMMA-UE revealed the strongest and significant correlation for curve efficiency (*r* = 0.75), followed by shoulder flexion/extension (*r* = 0.68), elbow flexion/extension (*r* = 0.53), and shoulder abduction/adduction (*r* = 0.49). Curve efficiency additionally correlated significantly with the arm subsection, focusing on synergistic control (*r* = 0.59).

**Conclusion:** The kinematic parameters of the upper limb after stroke were influenced largely by the task. These results underpin the necessity to assess different relevant functional movements close to real-world conditions rather than relying solely on clinical measures.

**Study Registration**: clinicaltrials.gov, identifier NCT03135093 and BASEC-ID 2016-02075.

## Introduction

Incidences of upper limb impairments after stroke have been reported in 48 to 85% of acute stroke patients (Jørgensen et al., 1999; Persson et al., 2012). Acute deficits might include paresis, ataxia, and loss of sensory function (Yew and Cheng, 2009). The course of recovery from these impairments varies from complete restoration to different degrees of compensatory adaptation (Levin et al., 2009; Bernhardt et al., 2017). Throughout the course, deficits in interjoint coordination have been described as a key feature in stroke-related dysfunctions that is characterized by the reappearance of primitive movement synergies and the presence of joint coupling (Krakauer and Carmichael, 2017). Interjoint coordination has been defined as the process to spatially and temporally arrange the degrees of freedom (DOF) needed to achieve the movement goal (Tomita et al., 2017) and is closely linked to the concept of synergies (Roh et al., 2013; McMorland et al., 2015; Santello and Lang, 2015). Based on two principal synergies, the flexor and the extensor synergy, pathological stereotypical coupling between two or more DOF has been observed as a phenotype of the loss of interjoint coordination after stroke. A loss of interjoint coordination is associated with weakness (Sukal et al., 2007) and spasticity (Allison et al., 2016) along the time course after stroke (Levin, 1996; Cirstea et al., 2003), leading to learned bad or non-use in daily life (Taub et al., 2006; Raghavan, 2015). Determining the level of interjoint coordination and associated motor dysfunction of stroke-related movement disabilities is critical to improve our understanding and expand interventional strategies to minimize long-term consequences due to stroke.

Interjoint coordination after stroke is often assessed by the Fugl–Meyer Motor Assessment of the Upper Extremity (FMMA-UE). This clinical assessment evaluates volitional movement control of the upper limb in a hierarchical manner from proximal to distal segments (Fugl-Meyer et al., 1975) and by taking into account the within-synergy, mixed-synergy, and out-of-synergy movement patterns as proposed by Twitchell (1951) and Brunnstrom (1966, 1970). Although the FMMA-UE has been attested to be of high quality in clinimetric properties (Gladstone et al., 2002), some limitations need to be considered in terms of the measurement construct being used. First, items of the FMMA-UE are assessed on a three-point ordinal scale (“not,” “partial,” and “fully”), and the “partial” category is very broad. An evaluation of “partial” movement achievement includes limitations in active range of motion or movement deviations, such as shoulder abduction or elbow flexion during shoulder flexion, that can range from small to exaggerated differences and cannot be differentiated further. This level of evaluation of movement quality does not allow to differentiate between physiological and pathological movement behavior (Kwakkel et al., 2017). Second, a full score in FMMA-UE cannot be directly related to complete restitution since deviations in movement kinematics and limitations in daily life might still be present (Thrane et al., 2019). Third, the FMMA-UE assesses mostly abstract movements and limb postures based on empirically derived stroke recovery stages that have little to no relevance to the subject's movements in daily life. Considering the widespread and recommended usage of the FMMA-UE as a primary outcome measure in stroke research trials (Santisteban et al., 2016; Burridge et al., 2019; Kwakkel et al., 2019; Subramanian et al., 2020) and the overall neutral results of most stroke rehabilitation trials (Corbetta et al., 2015; Eraifej et al., 2017; Veerbeek et al., 2017), the question on how far this outcome can sensitively capture changes on the body function level when performing daily life tasks cannot be omitted.

The introduction of modern technology opened new avenues for assessments of motor function. Upper limb kinematic motion analysis in the stroke population has been performed with 2D and 3D set-up conditions for assessing a large number of different kinematic outcome parameters in predominantly pointing or reach-to grasp tasks (Schwarz et al., 2019b). Kinematic parameters measure body functions and thereby characterize aspects of movement control, such as interjoint coordination. Outcome measures to quantify upper limb interjoint coordination include spatial measures of active range of motion in shoulder and elbow and of trunk displacement (van Kordelaar et al., 2012) that have been attested to be of sufficient validity and reliability in 3D pointing tasks (Subramanian et al., 2010; Massie et al., 2011, 2014; Wu et al., 2014). Measures of interjoint coordination, relating at least two DOF, ranged from angle–angle plots (Beer et al., 2007; Woodbury et al., 2009; Alt Murphy et al., 2011), correlation analysis (Yang et al., 2017), slope statistics (Baniña et al., 2017), and ratio or index measures (Cirstea and Levin, 2007; Levin et al., 2016) to mathematically more complex parameters, such as functional Principal Component Analysis (van Kordelaar et al., 2013) or approximate entropy metrics (Sethi et al., 2017). Parallel to this, movement timing or workspace measures, such as circle size area (Sukal et al., 2007; Krabben et al., 2011; Ellis et al., 2016), provide indirect measures as a result of pathological synergies. Taken together, the variety of metrics identified for evaluating interjoint coordination illustrate the wide context and aspects of this movement construct and tight connection between the movement characteristics and the chosen metric as, for example, the circle size area in a circle drawing task (Houwink et al., 2013). Considering this state-of-the-art in upper limb kinematic assessments, it could be proposed that research on interjoint coordination would profit from task-independent metrics that could be evaluated in various tasks and settings, thereby allowing for comparability, especially for pooling in meta-analysis.

In this study, first, it was questioned whether kinematic parameters representing aspects of interjoint coordination in the shoulder–elbow–trunk complex are different with respect to different movement tasks and the arm being tested by considering the dominant affected side, the upper limb function, and age as covariates. Second, it was examined whether statistically significant correlations can be found between each of the kinematic parameters of the affected side, the FMMA-UE full score, and the FMMA-UE arm subscale that evaluates the shoulder–elbow–trunk complex according to the synergy concept. The findings will provide new insights into the characteristic interjoint coordination in different functional and non-functional upper limb movements after stroke, propose kinematic parameters to quantify spatiotemporal aspects of interjoint coordination, and, as a long-term goal, support the establishment of feasible and repeatable qualitative kinematic assessments in close relation to real-world functional activities.

## Materials and Methods

A prospective cross-sectional study was performed at the rehabilitation clinic Cereneo, Vitznau, Switzerland, to explore the relationship between upper limb function and activity as measured by clinical assessments and by a wearable motion capture system. The study protocol was approved by the Cantonal Ethics Committee Northwest and Central Switzerland (BASEC-ID: 2016-02075) and prospectively registered in ClinicalTrials.gov (NCT03135093). Between July 2017 and October 2019, 523 patients from the stroke research register of the Department of Neurology, University Hospital Zurich (Zurich, Switzerland) were screened by telephone and onsite screening.

### Study Participants

The subjects were deemed eligible when they met the following inclusion criteria: (1) >6 months post-unilateral stroke (hemorrhage or ischemic), (2) at least 18 years of age, and (3) upper limb motor impairment, but at least partially able to lift the arm against gravity (>30° of shoulder flexion) and to flex and extend the fingers for basic grasping. The exclusion criteria were (1) an increased upper limb muscle tone with limitations in range of motion [modified Ashworth Scale (MAS) ≥3], (2) severe sensory deficits in the upper limb [Erasmus modifications to the revised Nottingham Sensory Assessment (EmNSA) of 0 in one of the test regions], (3) a preexisting orthopedic or neurological disease affecting movements of the upper limb, (4) contraindications on ethical grounds, e.g., persons who are decisionally impaired, (5) known or suspected non-compliance, or (6) severe communication or cognitive deficits that cause an inability to follow the study procedures as determined by the Montreal Cognitive Assessment (MoCA) ≤20 points (Dong et al., 2013). The MAS (Bohannon and Smith, 1987) and the EmNSA (Stolk-Hornsveld et al., 2006) were performed with the participant in supine position. The EmNSA was evaluated for the surface, pinprick, sharp-blunt, and proprioceptive discrimination in both arms. Each participant gave a written informed consent according to the Declaration of Helsinki and the Swiss regulatory authorities.

### Study Experiments

An experienced research therapist performed all the study experiments during a single-day measurement at the rehabilitation clinic Cereneo (Vitznau, Switzerland). The study experiments started after onsite screening and informed consent with setting up the wearable kinematic measurement system. When being acquired with the system, the participant performs the FMMA-UE and a set of daily living activities with both upper limbs. The less-affected side was assessed to determine the close-to-physiological-movement behavior on the best available level in delineation to pathological movement behavior of the affected upper limb during functional and non-functional activities.

### Measurement System

**Figure 1:**
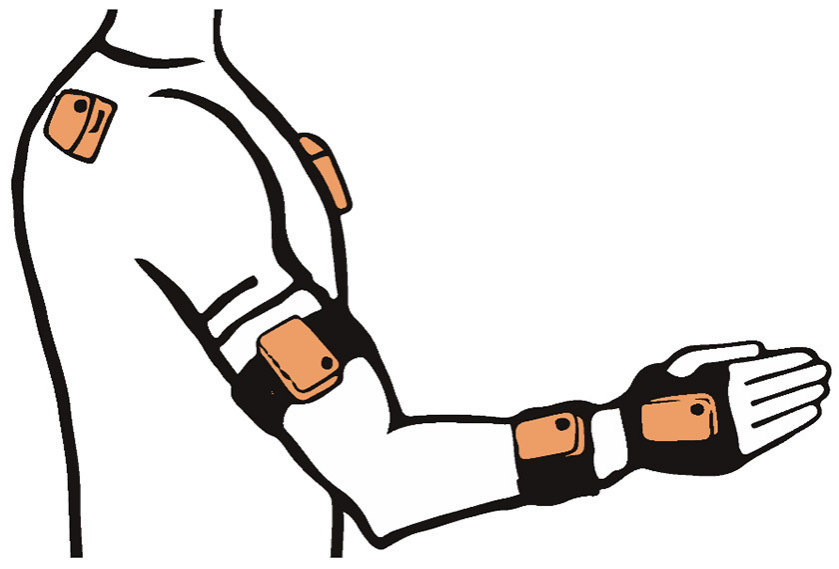
Measurement system set-up.

A portable and wireless sensor-based motion capture system was used to capture upper limb kinematics (Xsens MVN Awinda, Xsens Technologies, the Netherlands). The system consists of 17 inertial measurement units (IMU), a receiver station, and attachment equipment (MVN Manual, 2018). The nine IMUs of the upper body used in this analysis were fixated on a T-shirt above both scapulae with the sensors' x-axes parallel to the spina scapulae and above the sternum, with the sensor aligned with the x-axis, as illustrated in Figure 1. The upper extremity IMUs were mounted with elastic Velcro straps on the upper arm above the lateromedial part of the humerus bone, around the distal radioulnar joint, three fingers above the wrist, and on the dorsal palm of the hand by the use of a palm glove or medical tape in case the glove was not fitting. Each IMU contains 3D linear accelerometers, 3D rate gyroscopes, 3D magnetometers, and a battery. Combined with information of the subjects' body measures into a biomechanical model, the data of 3D angular velocity, 3D acceleration, 3D earth magnetic field, and atmospheric pressure allow as **Table 3D** orientation for human kinematic motion analysis (MVN software, 2018). The kinematic data are sampled at 60 Hz. The accuracy of the system to measure each body segments' position has been reported as ~5 mm and the orientation with a measurement error of 3° (Roetenberg et al., 2007a,b). The system was previously validated with a camera-based system (Optotrak) demonstrating comparable results (Robert-Lachaine et al., 2017) and additionally investigated for intra- and interrater reliability with fair to excellent results, even when being used by clinicians with no experience in applying motion capture technologies (Al-Amri et al., 2018).

Setting up the system for each participant included taking body measures, such as body height, shoulder height (distance from the ground to the top of the acromion), and shoulder width (distance between the right and the left lateral border of the acromion), the sensor attachment, and a calibration procedure that consisted of standing in neutral position at the calibration spot, walking 3 m, and returning to the start. The whole procedure took about 15 to 20 min and was completed when the subject returned back to neutral pose standing at the calibration spot. The measurements of all subjects were performed in an upright sitting position on an armless chair in the same examination room of the rehabilitation clinic, as well as the position and orientation of the subject. This allowed to control for possible external inferences that could affect the sensor data of the IMUs, such as electric leads.

### Movement Tasks

The selected movements consisted of four different discrete movement tasks: (1) isolated shoulder flexion, (2) pointing ahead, (3) reach-to-grasp a glass, and (4) key insertion into a lock. The selection was based on the shared upper limb workspace along the sagittal plane and discrete reaching movement while discriminating variations in non-functional and functional movements with and without grasp contact in alliance with existing upper limb movement (Schambra et al., 2019) and grasp taxonomies (Feix et al., 2016). An overview of the movement tasks including characteristics such as contact and the underlying motion primitives is provided in Table 1. Each movement task was demonstrated and instructed verbally, including demo-trials if necessary. The movement start was defined by a flick on one of the sensors. After task completion, the subjects were asked to return to the start position. For the analysis, the maximum shoulder flexion angle and/or the maximum distance of the hand IMU positional data along the x-axis defined the movement end. The chair had a standard seat height of 46 cm, with a back support 51 cm in height and with a backward inclination that was counterbalanced by fixing a tight pillow at the back of the chair. The table was height-adjustable to allow a subject-specific set up of 0° in all axis of the shoulder, 90° of elbow flexion, and with the hand pronated on the table. The subjects were instructed to perform the task at a comfortable speed while keeping contact with the back of the chair. This instruction was given once at the beginning to not interfere profoundly with the natural movement behavior. Three to six repetitions were performed with each upper limb to include at least three successful trials in the data analysis (Alt Murphy et al., 2018), starting with the less-affected side and followed by the affected side.

**Table 1:** Upper limb movement task characteristics.

**Task number**	**(1)**	**(2)**	**(3)**	**(4)**
Task name	Shoulder flexion	Pointing ahead	Reach-to-grasp a glass	Key insertion
Set-up Start position 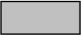 End position 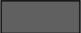	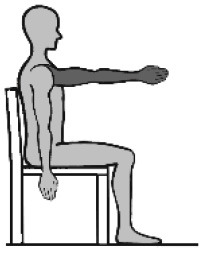	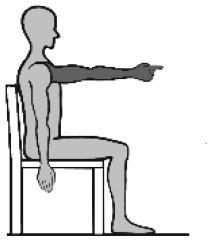	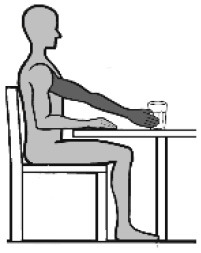	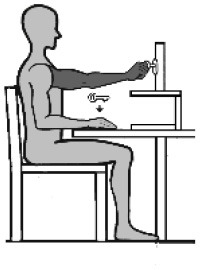
Task purpose	Non-functional	Functional	Functional	Functional
Description	According to FMMA-UE, item shoulder flexion from 0° to 90°, elbow in 0° extension and neutral forearm	Gesture of pointing in the air at shoulder height, to look ahead/ indicate to a visual scene in the distance	Reach to a non-filled glass placed in 90% arm's length, move it to the mouth, take a sip, and place it back	Pick up a key placed at the medial side of the subjects' hand, take it up, and insert the key into a lock on a top shelf (28.5cm) in 90% arm's length
Upper body effector	Proximal (shoulder, elbow)	Distal (index finger)	Distal (hand, finger)	Distal (MCP, thumb)
Movement focus	Internal	External	External	External
Contact type	No contact	No contact	Grasp contact at end	Grasp contact from start to end
Grasp type	Not applicable	Not applicable	Cylindrical grasp	Palm opposition
Functional motion primitive	Not applicable	Reposition or reach-to-point	Reach-to-grasp	Transport and stabilize
Movement phase for analysis	From movement start to maximum shoulder flexion (90°)	From movement start to maximum shoulder flexion	From movement start to grasp the glass	From key pick-up to insertion into lock

*FMMA-UE, Fugl–Meyer Motor Assessment of the Upper Extremity; MCP, metacarpo-phalangeal joint*.

### Outcome Measures

The recorded kinematic measures were segmented by movement trial based on the flip signal and the maximal target angle and stored in mvnx files for data transferring and processing in MATLAB (The MathWorks Inc., Natick, MA, USA). For each movement task, participant, and tested upper limb, the shoulder and elbow angles and the positional data of the trunk sensor of all repetitions were extracted for analysis. The kinematic parameters of interest consisted of spatial and spatiotemporal measures.

#### Spatial Parameters

Spatial measures included joint angle ranges in degrees around one rotation axis and trunk displacement in millimeter. Each joint can be expressed in six DOF around the orthogonally arranged rotation axis, where one joint angle is defined by a joint rotation as the orientation of a distal segment with respect to a proximal segment. Joint rotations are calculated using the Euler sequences ZXY and XZY by the MVN software (MVN Manual, 2018) based on the coordinate system agreed by the International Society of Biomechanics (ISB) (Wu et al., 2005). All angles follow the ISB Euler angle extractions of Z (flexion/extension), X (abduction/adduction), and Y (internal/external rotation), except for the shoulder joint where the Euler sequence XZY is used. The definitions of the origins of the segments are somewhat different from marker-based recommendations since MVN uses a motion tracker placed on the segment rather than markers placed on bony landmarks close to the joint origin (MVN Manual, 2018).

The range of motion was defined by calculating the minimum and maximum angle for all data points from movement onset to end (van Meulen et al., 2015). The standard deviations of the minimum and maximum joint angle were calculated as a measure of variability. For the purpose of this study to evaluate interjoint coordination in the shoulder–elbow complex, shoulder flexion/ extension, shoulder abduction/adduction, and elbow flexion/extension were captured and analyzed. Even though shoulder rotational movements are an important component of the upper limb, they were not considered in this study since the measurement accuracy of rotations around the transversal plane were associated with the largest measurement error ranging from 16° to 34° (Walmsley et al., 2018). The challenge to measure rotational movements on the transversal plane might be related to the larger differences between soft tissue and bone motions during rotation. Elbow flexion/extension was determined by rotation around the z-axes, where elbow extension was represented by 0° and positive values indicating flexion of the elbow. Shoulder flexion–extension was defined as an elevation parallel to the sagittal plane and angles that rotate around the z-axis. Shoulder abduction–adduction was defined as an elevation on the frontal plane and rotates around the x-axes of the shoulder joint. Positive values indicate shoulder flexion or abduction, and negative values indicate shoulder extension or adduction.

In contrast to ISB descriptions of the shoulder with the thorax, clavicle, scapula, and humerus, the MVN model does not define the thorax segment nor the clavicle. The MVN model splits the thorax region into spine segments (MVN Manual, 2018). In alliance with other studies in the field, trunk motions were simplified to trunk displacement as defined by changes in position and orientation of the sternum sensor between movement onset and end (Subramanian et al., 2010). The change in trunk displacement was calculated by subtracting the mean of the first 10 data points from the other position values in the x-, y-, and z-direction and were summarized by:

$$\text{Trunk displacement}=\sqrt{\left(\text{T}{\text{x}}^{2}+\text{T}{\text{y}}^{2}+\text{T}{\text{z}}^{2}\right)}$$

where ***Tx*** includes frontal displacement, ***Ty*** includes sideway displacement, and ***Tz*** includes displacement in rotation.

#### Spatiotemporal Parameters of Shoulder–Elbow Coordination

**Figure 2:**
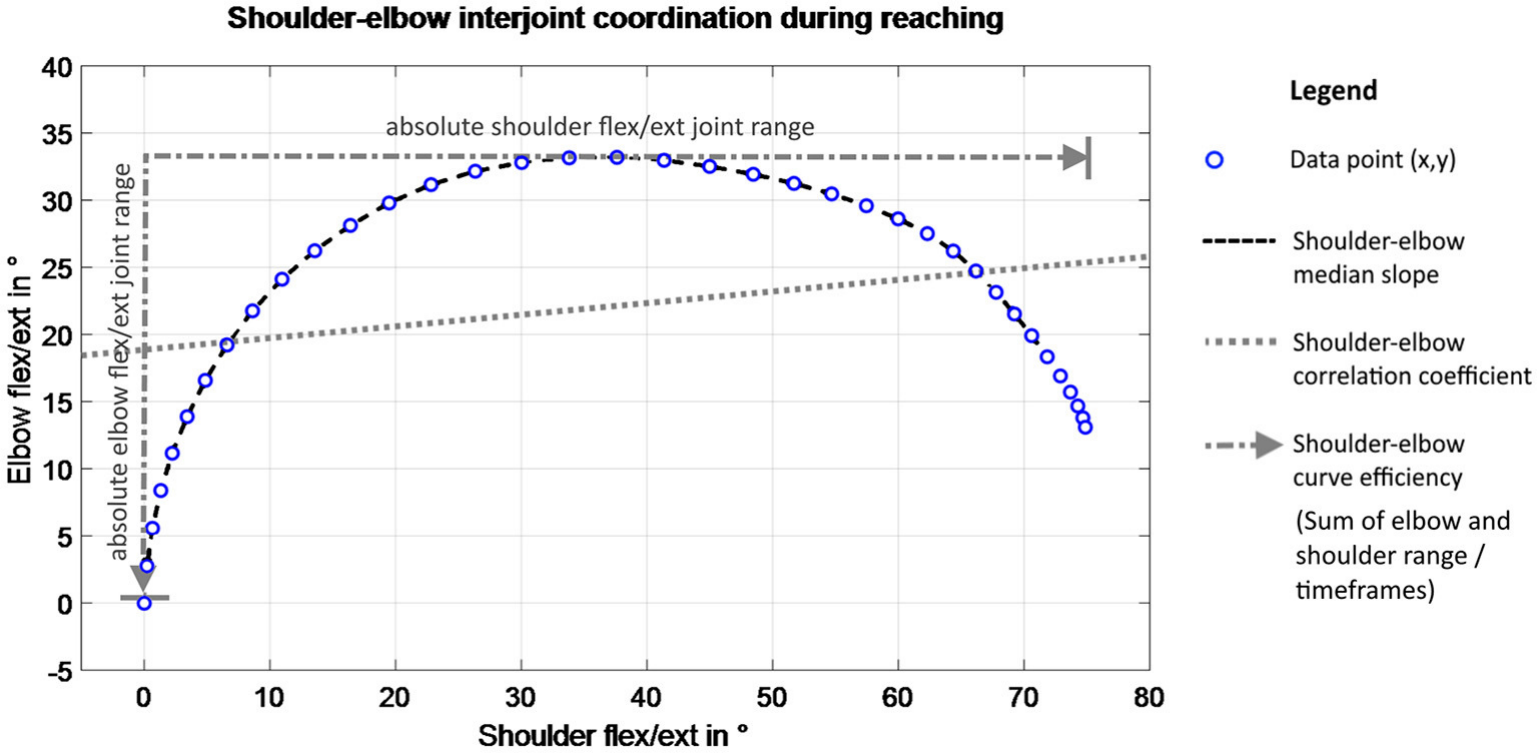
Schematic of shoulder–elbow coordination measures.

Angle–angle plots of the shoulder and the elbow flexion angle for each timeframe of the movement were derived to qualitatively analyze interjoint coordination and coupling between shoulder and elbow flexion/extension in reaching, as illustrated in Figure 2. For each movement repetition per participant, the elbow and shoulder angles were set to 0° or 90° according to the related starting position and time normalized with respect to the mean trial length to enable comparability.

A shoulder–elbow correlation coefficient was calculated to quantify the relationship between shoulder flexion/extension (SF) and elbow flexion/extension (EF) in the following equation:

r=∑m∑n(SFmn−SFmean)(EFmn−EFmean)(∑m∑n(SFmn−SFmean)^2∑m∑n(EFmn−EFmean)^2)

where *SF*_*mean*_ = *mean*(*SF*) and *EF*_*mean*_ = *mean*(*EF*).

In the case of isolated joint movements, a low correlation coefficient highlights the ability to uncouple joint movements, whereas a coupling relationship was detected if the change in movement direction of two segments occurred at the same time. In isolated joint motions of task (1), a well-coordinated movement with a constantly extended elbow would result in a correlation coefficient close to 0, whereas pathologically coupled movements would result in a higher correlation coefficient, according to the hypothesis of voluntary joint control. Reaching out for an object on a table is likely to start from an elbow flexed position and then requires the elbow to extend while the shoulder is being elevated so that a negative correlation would be expected for physiological movement and conversely a low correlation in case of pathological coupling with remaining elbow flexion while reaching out.

Shoulder–elbow median slope was defined by the slopes connecting the data points of elbow–shoulder angle–angle plots as depicted in Figure 2. The mean slope between elbow flexion/extension and shoulder flexion/extension was used to assess interjoint coordination by Baniña et al. (2017). In this present study, the median slope was selected instead of the mean slope to account for the non-linearity of angle–angle curves, especially in task (2), (3), and (4). The slope changes between the shoulder and elbow per timeframe ranges from positive to negative infinite values, representing the gradient of the curve.

Shoulder–elbow curve efficiency is included to quantify the maximum movement execution in the target DOF for the movement. It was defined by the sum of absolute joint range in shoulder flexion/extension and elbow flexion/extension, as visualized in Figure 2, divided by the number of data points of the movement to quantify the amount of both joint ranges in reaching. The sum of absolute joint ranges was normalized with respect to the number of frames to include the temporal efficiency of the movement. For isolated joint movements, such as in task (1), the absolute range in elbow flexion/extension is subtracted from the absolute range in shoulder flexion/extension, divided by the number of timeframes. For the other movement tasks, the absolute ranges in elbow and shoulder flexion/extension were summed up to quantify the upper limb movement magnitude during reaching. Values are given in degrees per frame, with higher values representing more efficient movement activation to reach the movement goal.

#### Clinical Measurements

The FMMA-UE was collected as a clinical stroke-specific measurement to evaluate upper limb motor impairment (Fugl-Meyer et al., 1975). The FMMA-UE is hierarchically composed, starting with assessing reflex appearance and primitive synergy patterns followed by within- to out-of-synergy movements in the arm subscale, based on the assumption that recovery “follows a definable stepwise course.” The FMMA-UE is partitioned into four sections, “upper extremity,” “wrist,” “hand,” and “coordination and speed,” as differences in recovery in each subscale could be independent from each other. Each test item is rated based on the best performance with the full FMMA-UE score ranging from zero to 66. For the purpose of this study, upper limb functionality subgroups were considered based on (Hoonhorst et al., 2015). who stratified FMMA-UE scores according to upper limb capacity measures that include grasping and displacement movements. With this subgroup selection, it was intended to investigate differences with respect to the subjects' capacity in grasping performance.

The information on hand dominance was obtained by asking the individual which hand he or she preferred to use for writing and throwing a ball prior to the stroke.

### Statistical Analysis

The statistical analysis was performed using Matlab (MATLAB version 2016b, The Mathwork, Natick, MA) and SPSS (SPSS version 26.0, IBM Corp., Armonk, NY, USA). Spatial measures of joint ranges in elbow flexion/extension, shoulder flexion/extension, and shoulder abduction/adduction were presented in absolute range of motion from minima to maxima with the corresponding standard deviations. Trunk displacement was given by absolute displacement from minima to maxima in millimeters. Spatiotemporal measures of shoulder–elbow coordination included the correlation coefficients *r*, the median slope, and the curve efficiency. All kinematic outcome parameters were explored for determining normal distribution in histograms and QQ plots. The descriptive statistics of the kinematic measures were summarized for all subjects and for each task and tested limb separately.

A linear mixed-model analysis was performed for each kinematic parameter to account for mixed effects in a repeated-measurement design. Each kinematic metric was treated as a dependent variable with respect to the independent fixed factors movement task (shoulder flexion, pointing, reach-to-grasp a glass, key insertion), the tested arm (affected, less-affected side), dominant hand is the affected hand (yes, no), the upper limb functionality group, as assessed with the FMMA-UE (32–47 points, “limited”; 48–52 points, “notable”; 53–66 points “full”) (Hoonhorst et al., 2015) and age (≤55 years and ≥56 years) (Kwakkel et al., 2017).

The relationship between clinically measured impairment and kinematic measures was examined by Spearman rank correlations. To evaluate the relationship between shoulder–elbow coordination, as measured in the FMMA-UE arm subsection when compared to spatiotemporal measures, Spearman rank correlation was used. According to the COSMIN guidelines, correlations between two measures of the same construct should be *r* ≥ 0.5, correlations of related measures *r* = 0.3–0.5, and correlations of unrelated constructs *r* < 0.3 (Prinsen et al., 2018). All statistical tests were performed at a significance level of 5%.

## Results

**Figure 3:**
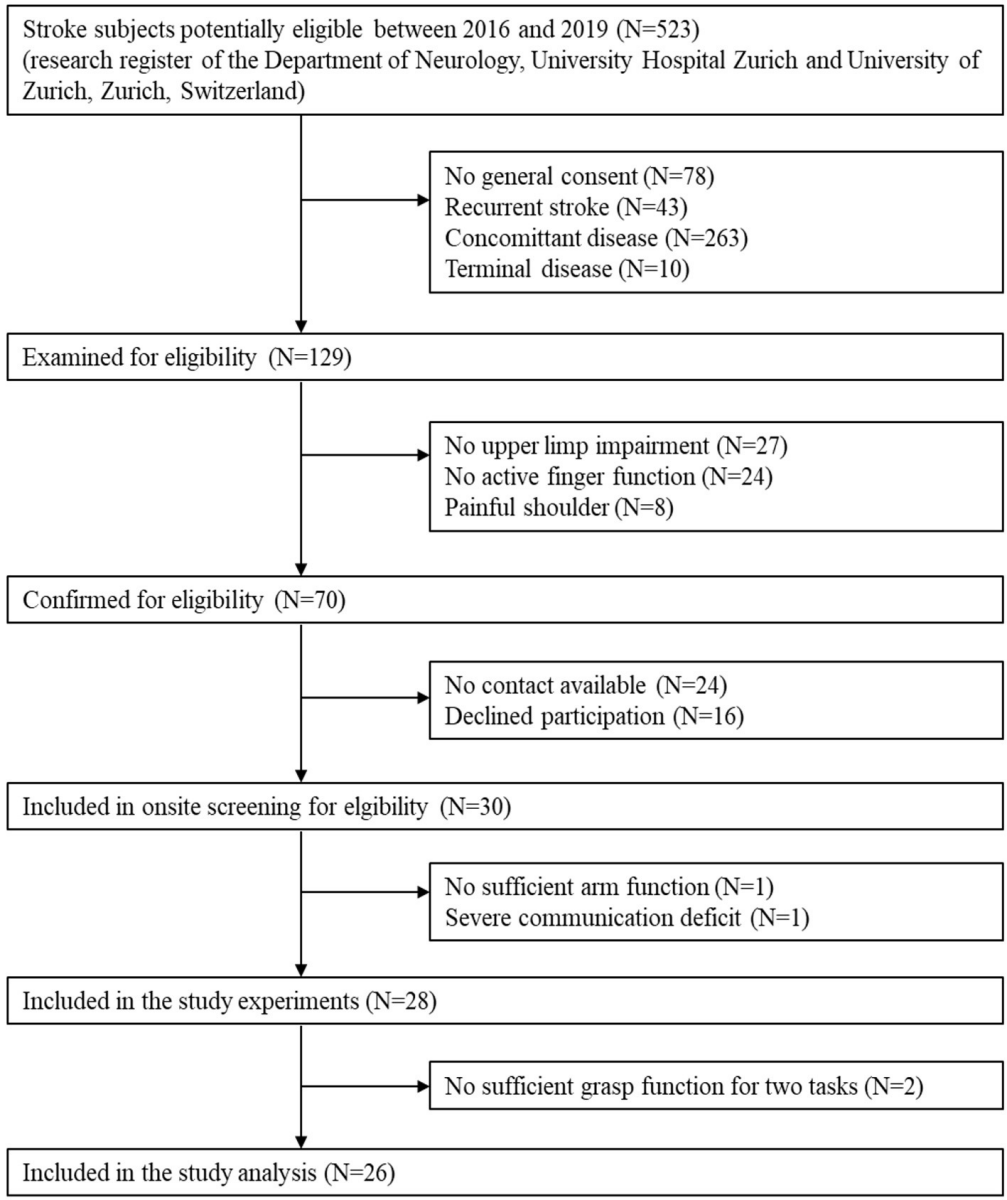
Study flow of the participants.

A total of 28 stroke subjects were included in the study, of which 26 were included in the data analysis. The study flow of participant inclusion is shown in Figure 3. The participant characteristics are summarized in Table 2. The study sample represents 26 mild to moderately impaired chronic stroke subjects, of whom 14 subjects were affected in their dominant upper limb. Seventeen subjects of the 26 included showed some resistance against passive movement in at least one of the tested muscles, as defined by a MAS score between 1 and 2. Sensory function was somewhat impaired in 21 subjects as determined by the EmNSA ranging from 29 to 40 points in the affected upper limb.

**Table 2:** Study participant characteristics.

**Characteristic**	**Total (*N* = 26)**
Gender, female/male	9/17
Mean age (SD), years	62.19 (12.10)
Mean body height (SD), cm	173.81 (10.94)
Mean BMI (SD), kg/m^2^	26.97 (4.23)
Paretic body side, left/right	13/13
Months since stroke^a^	20.50 (12–34)
Initial stroke severity NIHSS^a^	8 (6–11)
MoCA (0–30)^a^	27 (24–28)
MAS sum of the upper extremity (0–14)^a^^b^	1.75 (0.25–3)
Shoulder internal rotator muscles (%)^b^	42.3
Biceps brachii muscle (%)^b^	69.2
Triceps brachii muscle (%)^b^	11.5
Wrist flexor muscles (%)^b^	23.1
Wrist extensor muscles (%)^b^	15.4
Finger flexor muscles (%)^b^	15.4
Finger extensor muscles (%)^b^	19.2
EmNSA-UE (0–40)^a^	38 (36–39)
FMMA-UE (0–66)^a^	47.50 (40.25–55.00)
FMMA-UE arm subsection (0–36)^a^	26 (22.00–29.75)
FMMA-UE wrist subsection (0–10)^a^	6 (6.00–7.75)
FMMA-UE hand subsection (0–14)^a^	11 (9.00–14.00)
FMMA-UE coordination subsection (0–6)^a^	4 (3.25–5.00)

*BMI, body mass index; EmNSA, Erasmus modified version of the Nottingham Sensory Assessment; FMMA-UE, Fugl–Meyer Motor Assessment of the Upper Extremity; MAS, modified Ashworth Scale; MoCA, Montreal Cognitive Assessment; NIHSS, National Institutes of Health Stroke Scale; L, left; SD, standard deviation*.

a *Values are presented in median (interquartile range)*.

b *MAS scores between 1 and 2 for the tested muscle*.

### Kinematic Characteristics per Movement Task of the Affected and Less-Affected Side

**Figure 4:**
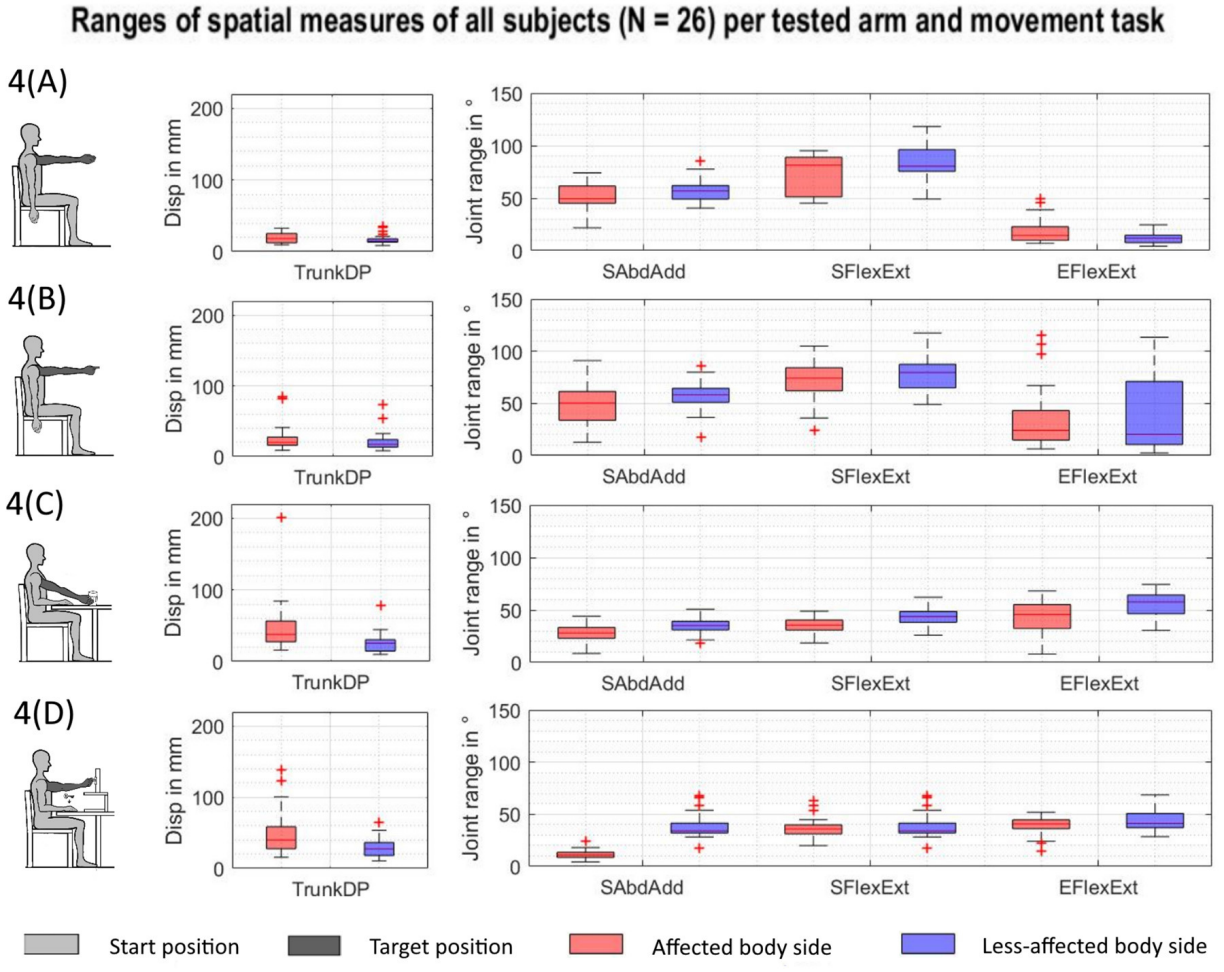
Spatial measures of the affected and less-affected arm per task across subjects (*N* = 26). AS, affected side; EFlexExt, elbow flexion/extension; LAS, less-affected side; SFlexExt, shoulder flexion/extension; SAbdAdd, shoulder abduction/adduction; TrunkDP, trunk displacement. **(A)** Shoulder flexion. **(B)** Pointing ahead. **(C)** reach-to-grasp a glass. **(D)** Key insertion.

Overall, 468 kinematic datasets per arm were included in the analysis, representing 26 stroke subjects, when performing four upper limb movement tasks. The observed QQ plots for the kinematic parameters did not lead to rejecting the assumption of normal distribution in the analyzed data. The spatial measures of joint ranges in elbow flexion/extension, shoulder flexion/extension, shoulder abduction/adduction, and trunk displacement are summarized for all subjects, each movement task, and affected (red-colored) and less-affected upper limb (blue-colored) in Figures 4A–D. Each boxplot illustrates the median, the upper and lower quartile, the minimum, and the maximum, as well as outliers shown as a red plus for each of the spatial measures. Different ranges across the spatial measures can be seen between the tasks. While increased trunk motions are shown in Figures 4C,D when compared to Figures 4A,B, shoulder flexion/extension shows larger ranges in Figures 4A,B when compared to Figures 4C,D.

**Figure 5:**
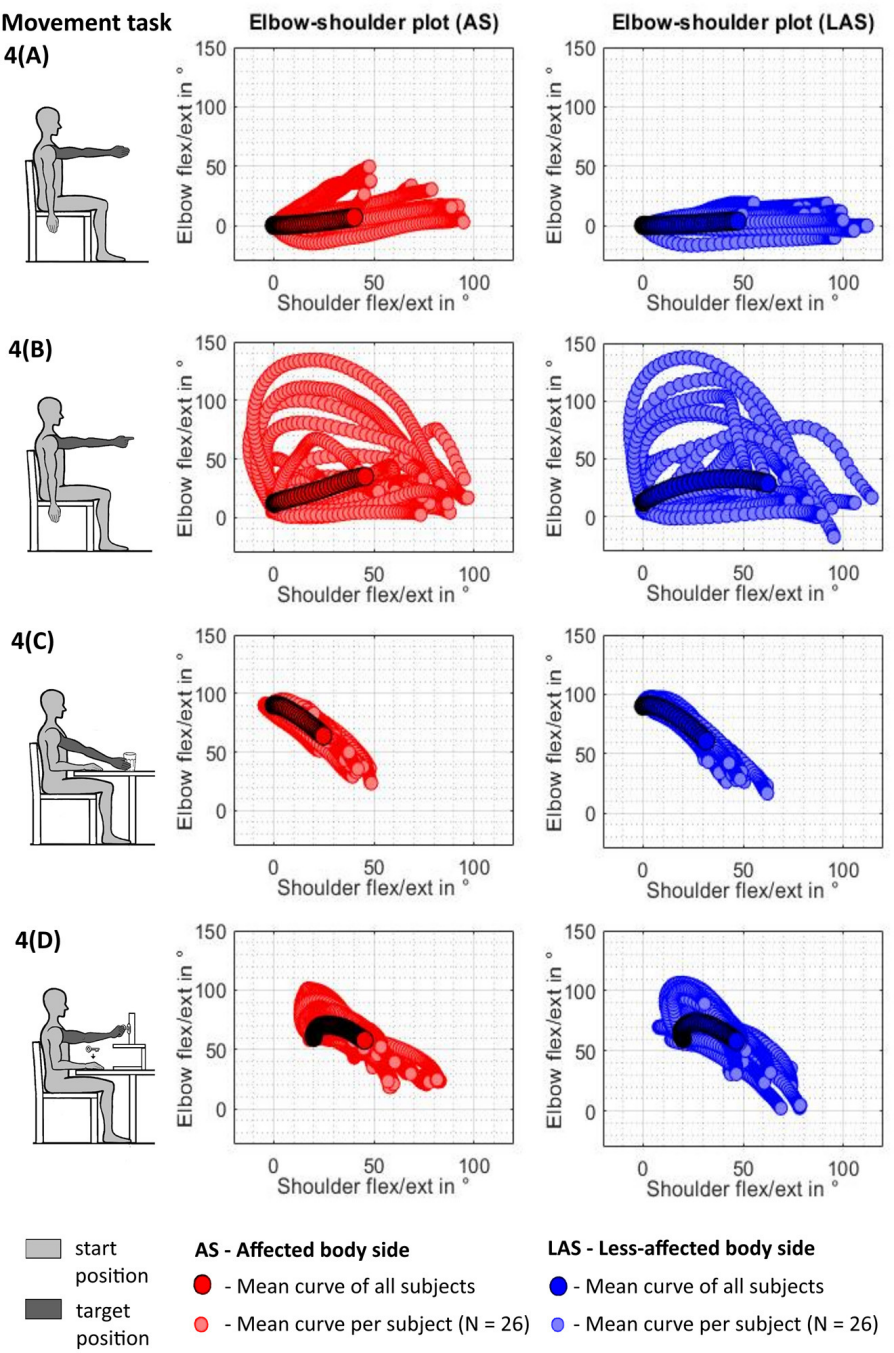
Shoulder–elbow mean curve per tested arm and task across subjects (*N* = 26). **(A)** Shoulder flexion. **(B)** Pointing ahead. **(C)** reach-to-grasp a glass. **(D)** Key insertion.

The spatiotemporal kinematics are illustrated in terms of shoulder–elbow angle plots for each movement task in Figures 5A–D. Each scatter curve represents the normalized mean curve per subject arm and task. Visual exploration of the shoulder–elbow angle plots depicts that deviations in terms of an increase of elbow flexion during shoulder flexion task (1) can be observed in all subjects and both arms while being increased in the affected upper limb in Figure 5A. Shoulder–elbow angle plots of the pointing ahead movement in task (2) revealed different movement strategies to emphasize the direction to look at between subjects in both the affected and less-affected upper limb. Figure 5B illustrates that subjects tended to either move through wide ranges of elbow flexion–extension, emphasize elbow extension at the end of the movement, or keep the elbow relatively extended throughout the movement. The shoulder–elbow angle plots of task (3) in Figure 5C illustrate comparable curve shapes during reaching in the affected and unaffected upper limbs. Similarly, curve shapes during task (4) in Figure 5D are comparable in both the affected and the unaffected upper limb. Besides the inter- and intra-individual movement variability, a preservation of the shoulder–elbow coordination can be described across the functional movement tasks when comparing the mean curve shape per subject of the shoulder–elbow plots between the affected side and the less-affected side.

### Effects of the Factors on the Spatial and Spatiotemporal Kinematic Measures

The mean estimates and standard deviation of the investigated kinematic parameter are presented for each fixed factor in Table 3. The results of the fixed-effects analysis per independent factor (task, tested arm, affected is dominant side, upper limb function, age) on each dependent kinematic measure are shown in Table 4. The results of *post hoc* pairwise testing between the four movement tasks and the three upper limb function levels are shown in terms of *p*-values per kinematic parameter and factor in Table 4.

**Table 3:** Descriptive statistics for each kinematic parameter per task and tested arm.

**Factor**	**TrunkDP in mm**	**SAbAd in°**	**SFleEx in°**	**EFlEx in°**	**SE Corr Coeff**	**SE median slope**	**SE curve efficiency in °/frame**
Movement							
Task (1) Task (2) Task (3) Task (4)	17.7 ± 6.9 20.1 ± 12.4 24.6 ± 13.1 29.7 ± 15.3	54.0 ± 8.4 53.6 ± 12.2 32.2 ± 6.1 17.7 ± 6.2	77.5 ± 13.5 74.2 ± 14.4 37.6 ± 8.0 36.9 ± 10.3	9.4 ± 7.5 40.3 ± 26.2 52.2 ± 10.8 41.5 ± 8.5	0.94 ± 0.1 0.33 ± 0.4 −0.96 ± 0.0 −0.75 ± 0.2	0.14 ± 0.1 −0.30 ± 0.7 −1.38 ± 0.2 −1.44 ± 0.4	0.7 ± 0.3 2.0 ± 0.6 1.5 ± 0.5 0.6 ± 0.3
Arm teste							
AS NA	24.8 ± 9.0 21.3 ± 12.4	34.4 ± 6.1 44.4 ± 6.6	53.2 ± 8.7 59.9 ± 8.9	36.9 ± 9.8 34.8 ± 11.2	0.10 ± 0.1 −0.11 ± 0.1	−0.64 ± 0.3 −0.85 ± 0.3	± 0.3 1.4 ± 0.4
Affected is dominant side							
Yes No	23.1 ± 10.7 22.9 ± 8.6	38.8 ± 6.5 39.9 ± 5.1	53.4 ± 11.6 59.7 ± 9.1	34.8 ± 11.2 36.9 ± 9.8	−0.10 ± 0.1 −0.11 ± 0.1	−0.77 ± 0.3 −0.72 ± 0.2	± 0.4 1.3 ± 0.3
UL function group							
Limited Notable Full	24.2 ± 9.2 24.7 ± 17.4 20.2 ± 9.4	38.7 ± 5.5 40.5 ± 10.8 39.0 ± 5.6	57.0 ± 9.8 50.8 ± 19.5 61.9 ± 10.1	34.9 ± 10.2 34.3 ± 16.1 38.2 ± 10.4	−0.10 ± 0.1 −0.09 ± 0.1 −0.12 ± 0.1	−0.72 ± 0.4 −0.83 ± 0.3 −0.69 ± 0.4	1.2 ± 0.3 ± 0.6 1.5 ± 0.4
Age group							
≤55 years >56 years	23.2 ± 12.1 22.8 ± 8.2	39.3 ± 7.4 39.5 ± 4.9	57.4 ± 13.3 55.7 ± 8.6	36.0 ± 12.2 35.6 ± 9.6	−0.10 ± 0.1 −0.11 ± 0.1	−0.78 ± 0.3 −0.71 ± 0.2	1.2 ± 0.4 1.2 ± 0.3

*Corr Coeff, correlation coefficient; EFlEx, elbow flexion/extension; full, full UL function (FMMA-UE 53-66); limited, limited UL function (FMMA-UE 32-47); notable, notable function (FMMA-UE 48-52); SAbAd, shoulder abduction/adduction; SFlEx, shoulder flexion/extension; TrunkDP, trunk displacement; SE, shoulder–elbow; UL, upper limb*.

**Table 4:** Results of linear mixed model analysis.

**Factor**	**Kinematic metric for interjoint coordination**						
	**TrunkDP**	**SAbAd**	**SFlEx**	**EFlEx**	**SE corr coeff**	**SE median slope**	**SE curve efficiency**
**Movement Task** Task (1) vs. Task (2) Task (1) vs. Task (3) Task (1) vs. Task (4) Task (2) vs. Task (3) Task (2) vs. Task (4) Task (3) vs. Task (4)	**0.001** 1.000 0.056 **0.002** 0.861 0.058 0.714	**<0.001** 1.000 **<0.001** **<0.001** **<0.001** **<0.001** **<0.001**	**<0.001** 1.000 **<0.001** **<0.001** **<0.001** **<0.001** 1.000	**<0.001** **<0.001** **<0.001** **<0.001** 0.129 1.000 **<0.001**	**<0.001** **<0.001** **<0.001** **<0.001** **<0.001** **<0.001** **<0.001**	**<0.001** **0.020** **<0.001** **<0.001** **<0.001** **<0.001** 1.000	**<0.001** **<0.001** **<0.001** 1.000 **0.001** **<0.001** **<0.001**
**Arm tested** AS vs. NA	**0.001**	**<0.001**	**<0.001**	**0.002**	0.395	**<0.001**	**<0.001**
**Affected is dominant side** (Yes vs. No)	0.935	0.413	**0.011**	0.251	0.089	0.161	**0.015**
**UL function group** Limited vs. notable Limited vs. full Notable vs. full	0.257 1.000 0.317 0.791	0.693 1.000 1.000 1.000	0.051 0.325 0.244 0.053	0.264 1.000 0.335 0.755	0.070 0.342 0.328 0.073	0.069 0.122 1.000 0.070	**0.001** 0.498 **0.003** **0.005**
**Age group** ≤55 years vs. >56 years	0.889	0.888	0.540	0.860	0.125	**0.045**	0.394

*Corr Coeff, correlation coefficient; EFlEx, elbow flexion/extension; full, full UL function (FMMA-UE 53-66); limited, limited UL function (FMMA-UE 32-47), notable, notable function (FMMA-UE 48-52); SAbAd, shoulder abduction/adduction; SFlEx, shoulder flexion/extension; TrunkDP, trunk displacement; SE, shoulder-elbow; UL, upper limb. Statistically significant effects (p < 0.05) are indicated in bold*.

Statistically significant differences were found for all movement tasks and all investigated kinematic parameters as displayed in Table 4. Trunk displacement ranged from 1.7 to 2.9 cm between tasks and was only statistically significantly different between isolated shoulder flexion and the key insertion task [*F*_(3, 58.036)_ = 6.119, *p* = < 0.001]. Effects of the factor of the tested arm were found for all kinematic parameters except of the shoulder–elbow correlation. The factor of affected dominant hand or affected non-dominant resulted in statistically significant effects on shoulder flexion/extension [*F*_(1, 39.832)_ = 7.058, *p* = 0.011] and shoulder–elbow curve efficiency [*F*_(1, 61.565)_ = 6.323, *p* = 0.015]. Differences with respect to upper limb function were detected for shoulder–elbow curve efficiency [*F*_(2, 61.565)_ = 7.285, *p* = 0.001], with significant differences between the limited (*N* = 13) and full function (*N* = 10) and between notable (*N* = 3) and full function in *post hoc* testing. The factor of age revealed significant effects on the dependent variable of shoulder–elbow median slope, with a mean of −0.784 compared to −0.705 in the less-affected side [*F*_(1, 34.432)_ = 4.344, *p* = 0.045].

### Relationship Between Clinically Measured Impairment and Spatiotemporal Kinematics

For the comparison between spatial and spatiotemporal kinematic measures across tasks per subject and the FMA-UE, correlation coefficients were calculated for each combination and presented in the confusion matrix in Table 5. The strongest statistically significant correlation with the FMA-UE was found for curve efficiency (*r* = 0.75), followed by shoulder flexion/extension (*r* = 0.68), elbow flexion/extension (*r* = 0.53), and shoulder abduction/adduction (*r* = 0.49). Furthermore, strong correlations were found between elbow flexion/extension and shoulder flexion/extension (*r* = 0.53), between elbow flexion/extension and shoulder abduction/adduction (*r* = 0.53), and between shoulder flexion/extension and shoulder abduction/adduction (*r* = 0.57). For shoulder–elbow curve efficiency, significant correlations were shown with shoulder flexion/extension (*r* = 0.85) and elbow flexion/extension (*r* = 0.55).

**Table 5:** Confusion matrix of the correlation coefficients for each measure combination.

	**TrunkDP**	**SAbAd**	**SFlEx**	**EFlEx**	**SE Corr Coeff**	**SE median slope**	**SE curve efficiency**
FMA-UE	−0.16 *p* = 0.436 (−0.51–0.24)	**0.49** **p = 0.010** **(0.13–0.73)**	**0.68** **p = 0.000** **(0.40–0.84)**	**0.53** **p = 0.004** **(0.19–0.76)**	0.10 *p* = 0.603 (−0.29–0.47)	0.053 *p* = 0.791 (−0.33–0.42)	**0.75** **p = 0.000** **(0.52–0.88)**
TrunkDP		−0.11 *p* = 0.595 (−0.47–0.28)	−0.12 *p* = 0.550 (−0.48–0.27)	−0.04 *p* = 0.831 (−0.42–0.34)	−0.10 *p* = 0.611 (−0.46–0.29)	−0.08 *p* = 0.678 (−0.45–0.31)	−0.04 *p* = 0.846 (−0.41–0.35)
SAbAd			**0.57** **p = 0.002** **(0.24–0.78)**	**0.53** **p = 0.004** **(0.19–0.76)**	−0.03 *p* = 0.880 (−0.41–0.35)	0.28 *p* = 0.154 (−0.11–0.60)	**0.40** **p = 0.040** **(0.02–0.68)**
SFlEx				**0.53** **p = 0.004** **(0.19–0.76)**	−0.20 *p* = 0.318 (−0.54–0.20)	−0.02 *p* = 0.921 (−0.40–0.36)	**0.85** **p = 0.000** **(0.70–0.93)**
EFlEx					−0.26 *p* = 0.198 (−0.58–0.14)	−0.15 *p* = 0.450 (−0.50–0.24)	**0.55** **p = 0.003** **(0.21–0.77)**
SE Corr Coeff						−0.07 *p* = 0.712 (−0.44–0.31)	−0.18 *p* = 0.380 (−0.52–0.22)
SE slope median							0.01 *p* = 0.956 (−0.37–0.39)

*The correlation coefficient, the p-value, and the 95% confidence interval are shown. The italicized measures present statically significant correlations*.

*EFlexExt, elbow flexion/extension; SFlexExt, shoulder flexion/extension; SAbdAdd, shoulder abduction/adduction; Trunk DP, trunk displacement; SE, shoulder–elbow; Corr Coeff, correlation coefficient; UL, upper limb. The bold measures present statistically significant correlations*.

**Figure 6:**
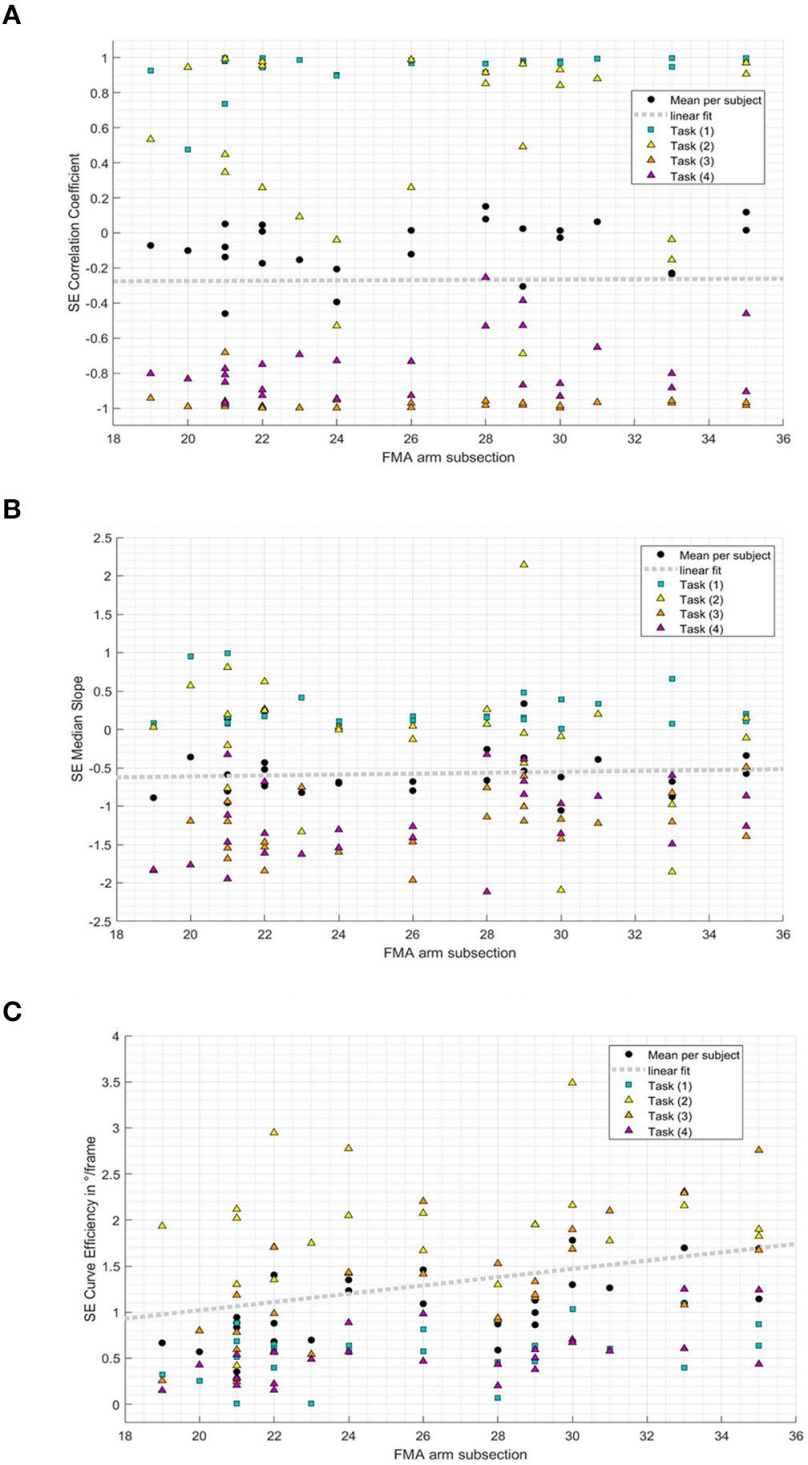
Relation between shoulder–elbow coordination metrics and the Fugl–Meyer Motor Assessment of the Upper Extremity arm subsection (19-35/36) per subject and task. **(A)** Shoulder-elbow correlation coefficient per subject FMMA-UE arm subsection and task. **(B)** Shoulder-elbow median slope per subject FMMA-UE arm subsection and task. **(C)** Shoulder-elbow curve efficiency per subject FMMA-UE arm subsection and task.

The relationship between the FMMA-UE arm subsection and kinematic metrics representing measures of shoulder–elbow coordination was additionally investigated to explore the comparability of kinematic measures and shoulder–elbow coordination as specifically tested in the FMMA-UE arm subsection. In the result, a statistically significant correlation between the clinically measured impaired interjoint coordination and curve efficiency (*r* = 0.59, *p* = 0.002) was found. For the shoulder–elbow correlation coefficient (*r* = 0.24, *p* = 0.230) and shoulder–elbow median slope (*r* = 0.09, *p* = 0.653), no statistically significant correlations were found with the FMMA-UE arm subsection. Figure 6 illustrates the subjects' mean values of the correlation coefficient, the median slope, and curve efficiency over all tasks and for each task plotted against the FMMA-UE arm subsection.

## Discussion

To our knowledge, this was the first study to investigate interjoint coordination during representative upper limb tasks in chronic stroke patients with mild to moderate upper limb motor impairment, aiming to bridge the gap between abstract clinical motor assessments and the kinematic characterization of various upper limb movements performed in daily life. Kinematic metrics reflecting interjoint coordination were investigated and compared across movement tasks by considering the covariates' dominance, age, and upper limb function and related with a recommended standard clinical test, the FMMA-UE (Kwakkel et al., 2017, 2019; Burridge et al., 2019). It was found that the values of kinematic metrics were largely dependent on the movement task and the tested arm, while age and the affected dominant side hardly influenced the metrics. The fact that both spatial and spatiotemporal metrics of the shoulder–elbow complex were largely dependent on the movement performed underpins the need to assess upper limb interjoint coordination in different task contexts. Interestingly, the elbow joint ranges were significantly different and less variable during isolated shoulder flexion task (9.4° ± 7.5°), representative for one of the FMMA-UE items, when compared to the pointing task (40.3° ± 26.2°), even though both tasks shared the same person-related workspace and target position, indicating the differences of the FMMA-UE from natural movement behavior. Comparing the results of the clinically measured impairment with the FMMA-UE and the resulting kinematic metrics across all tasks revealed moderate correlations between the FMMA-UE or FMMA-UE arm subsection and metrics on shoulder and elbow joint ranges and shoulder–elbow curve efficiency (*r* ≥ 0.5) besides low correlations between trunk metrics and shoulder–elbow correlation coefficient and median slope.

All spatial and spatiotemporal kinematic measures, except the shoulder–elbow correlation coefficient, showed statistically significant discriminability between pathological movement behavior of the affected upper limb and physiological movement behavior of the less-affected upper limb.

Trunk motions ranged between a mean of 1.8 and 3.0 cm, tending to increase from the shoulder flexion, pointing ahead, reach-to-grasp a glass, to the key insertion task. This illustrates increased trunk compensation, with an increase in task complexity by requiring distal upper limb interactions with objects (McIsaac et al., 2015). Trunk compensatory movements were shown to be slightly but significantly increased when moving the affected limb (mean of 2.5 cm) when compared to the less-affected upper limb (mean of 2.1 cm). However, these differences were small when compared to previous findings of trunk movements of around 10 cm in stroke subjects during reach-to-point (Cirstea et al., 2003) and reach-to-grasp (Alt Murphy et al., 2018). Hence, the presented results fall within the limits of 2 to 5 cm as a clinically meaningful cutoff score for compensatory trunk movements (Alt Murphy et al., 2013). The differences in the shoulder DOF can be partially explained by differences in target height between tasks, especially between the reach-to-grasp a glass on the table that requires less shoulder flexion when compared to the other movement tasks with targets on shoulder-height level. Shoulder joint ranges in flexion/extension and abduction/adduction were diminished in the affected arm in comparison to the less-affected arm, with joint ranges of 53° vs. 60° and 34° vs. 44°, respectively, suggesting inefficient activation or weakness of the shoulder muscles and the inability to cope with antigravity torques (Roh et al., 2013). Elbow flexion/extension ranged from a mean of 9.4° in isolated shoulder flexion and around 52° during functional task execution. The larger ranges in elbow flexion/extension during functional movements when compared to non-functional isolated shoulder flexion support the idea, that the elbow joint is rather dynamically involved in reaching movements of daily life activities than being involved as a stable or stabilizing component of a movement as predominantly examined in the FMMA-UE.

On the level of spatiotemporal measures of shoulder–elbow coordination, values of the correlation coefficient largely varied between *r* = −0.9 and *r* = 0.9 within and between subjects with a tight connection to the movement tasks as illustrated in Figure 4. The correlation coefficient is a measure of the linear relationship between two variables, such as shoulder flexion/extension and elbow flexion/extension. Although the correlation coefficient provides estimates of general trend between two variables, it does not consider non-linearity in rather bell-shaped angle–angle curves. The shoulder–elbow median slope represents estimates of the relationship between two DOF per timeframe (Baniña et al., 2017). Both the correlation coefficient and the median slope are quantifications of the overall trend in the shoulder–elbow curve and depend on both the type of movement as well as whether the shoulder and elbow move inphase or outphase. Consequentially, both metrics are limited to the general relationship between two joints. Shoulder–elbow curve efficiency ranged between a mean of 0.14° and 1.44° per frame with respect to the movement task. Curve efficiency was considerably lower in the shoulder flexion and key insertion task when compared to the other tasks, which could be an indicator of the increased requirements on movement preciseness during key insertion and increased internal attentional focus during isolated shoulder flexion. Curve efficiency was introduced as a novel measure of interjoint coordination that combines the absolute spatial changes in two DOF while considering temporal aspects in terms of timeframes needed to perform the movement. In that sense, curve efficiency accounts for the proposed definition of interjoint coordination by Tomita and coworkers as “a goal-oriented process in which the DOF are organized in both spatial and temporal domains such that the body configuration enables the endpoint to reach to a desired location in a context dependent manner” (Tomita et al., 2017). Herein curve efficiency has proven to be discriminable with respect to the factor whether the affected hand is the dominant hand and with respect to the upper limb motor function group, indicating promising associations with upper limb motor impairment levels.

Taken together, these findings confirm the importance of including different upper limb movement tasks when looking at interjoint coordination in patients after stroke and non-disabled adults as the task strongly affects kinematic metric outcomes (Jeannerod, 1990; Michaelsen et al., 2004; Mesquita et al., 2020). Adding up to these task-related kinematic differences, research on functional brain activation provides evidence that the cerebral control of upper limb movements is arranged in a task-specific action topography by taking the activity as a whole rather than being controlled by separating or combining movement components or specific or fixed brain areas (Handjaras et al., 2015; Leo et al., 2016). The findings of the present study emphasize the importance to consider the effects of the movement purpose, the attentional focus, and the movement complexity on kinematic expressions complementary to clinical assessment evaluations. Unlike the shoulder flexion movement of the FMMA-UE that relies on an internal movement focus and a stable extended elbow position, the three representative functional tasks rely on an external movement focus with mainly inverse kinematics between shoulder flexion and elbow extension and bell-shaped angle–angle profiles. Even though further curve fitting analysis is out of the scope of the present study, a visual inspection of the shoulder–elbow angle plots suggests that motions in the shoulder and elbow were diminished in the affected side when compared to the less-affected side, while the task-associated shapes seem to be largely preserved in the affected limb. These results furthermore underpin the challenge to clearly distinguish pathological from physiological interjoint coordination and movement activation in terms of active range of motion and strength at least in natural surroundings, including the constant influence of gravity.

The relationship between the spatiotemporal kinematic measures and the clinically measured upper limb motor impairment was explored as a part of validity. The herein presented findings on correlation between the FMMA-UE and spatial metrics shoulder flexion/extension and elbow flexion/extension are in line with research on validity (Massie et al., 2011, 2014; Finley et al., 2012; van Kordelaar et al., 2012; de Paiva Silva et al., 2014; Li et al., 2015; Rech et al., 2020). In contrast to existing research (Subramanian et al., 2010; Finley et al., 2012; van Kordelaar et al., 2012; de Paiva Silva et al., 2014; Massie et al., 2014), we did not find a strong correlation between trunk displacement during various tasks and the FMMA-UE total score. The strong correlation between curve efficiency and shoulder flexion/extension and elbow flexion/extension found in this study might be related to the fact that curve efficiency is a derivative of both DOF besides the temporal aspect of this movement parameter. The fact that shoulder–elbow curve efficiency significantly correlated with the FMMA-UE arm subsection supports the idea that it measures the same construct of interjoint coordination in the upper extremity. Future work on upper limb kinematic measurements after stroke should investigate its clinimetric properties, such as reliability, measurement error, and responsiveness.

### Limitations

The spatiotemporal kinematic analysis of this study was limited to three out of seven DOF, namely, shoulder flexion/extension, shoulder abduction/adduction, and elbow flexion/extension, even though rotational movements and the forearm and hand component are known to be part of movement quality. We decided to examine interjoint coordination on the basic level of the two joints that contribute most to the movement performance and present characteristic stroke-related movement phenotypes, such as the pathological flexor synergy.

Another limitation relates to the fact that we have considered the less-affected upper limb as the physiological movement comparator, even though we were aware of the evidence on movement limitations in the ipsilesional upper limb (Bustrén et al., 2017). Nevertheless, the less-affected upper limb represents a valuable comparator in the asymmetrical impairment of unilateral stroke and is always available to the patient and the assessor in clinical practice (Lang et al., 2017). For this reason, comparisons between the affected limb and the less-affected upper limb remain the best-available comparator in terms of movement quality measures until a reasonable amount of normative kinematic data from the healthy population is available.

We have not controlled for possible strength limitations and therefore were not able to differentiate between weakness and interjoint coordination in the presented experimental set-up, as gradually studied by by Dewald and colleagues (Sukal et al., 2007; Ellis et al., 2016). This could be induced by including a gradual armload increase during movement task execution. Apart from that, real-world upper limb functions are performed not only in sitting but also in other body positions, such as standing. The fact that the pioneering works on interjoint coordination and synergistic control after stroke emphasized the influence of the postural setting of the subject on synergistic control (Fugl-Meyer et al., 1975) supports further research on this topic and its consideration in upper limb assessments.

In the current study, a wearable inertial sensing suit was used, and this goes against recent recommendations to capture upper limb kinematics by an opto-electronic device (Kwakkel et al., 2019). However, the pros of wearable sensing suits are the wide applicability in flexible environments, the avoidance of problems with marker occlusion during object manipulation, and the comparably less time-consuming system set-up (pre- and post-processing) and costs of the equipment (Walmsley et al., 2018). Based on previous research, the reliability and measurement error has shown to be comparable between inertial sensing and optoelectronic system (Robert-Lachaine et al., 2017), even when the system was used by an unexperienced person (Al-Amri et al., 2018).

Lastly, it needs to be acknowledged that other analytical approaches on the kinematic data, such as dimension–reduction approaches, would have been possible, allowing the presentation of other kinematic outcomes (Schwarz et al., 2019a). Kinematic measures of the movement smoothness domain have been used for quantifying interjoint coordination based on accelerometer or gyroscope signals in the lower limb during gait assessment (Beck et al., 2018) and should be additionally considered in future work of upper limb interjoint coordination besides the herein proposed measures.

### Future Research

Future research should expand on an upper limb movement task set (Kwakkel et al., 2019), allowing to assess the widest possible range of the tested subjects' functional capabilities by considering a stepwise increase of movement task complexity, task instruction, and focus (McIsaac et al., 2015). Including a functional planar task on the table level, such as wiping or shape-drawing, besides gesture movements, reach-to-grasp, and manipulating activities should be considered in such a task set and future works to enable kinematic evaluations in stroke subjects with lower levels of upper limb function and reducing load on the shoulder. Dual-task conditions should be included in the highest level of task difficulty to assess the functional capability under real-world conditions, for example, when cooking and talking at the same time, as well as to uncover subliminal deficits that still might impact the person's performance level in daily life. Another important aspect in upper limb assessments reflecting needs of real-world use is the consideration of posture. In this line, it would be interesting to investigate the impact of posture on upper limb kinematics. The resemblance with daily life tasks in such an assessment protocol is likely to ease task understanding and naturalness of the performance even in subjects with difficulties in understanding.

## Conclusions

The presented work on qualitative upper limb movement analysis confirmed that kinematic measures of interjoint coordination in the shoulder–elbow–trunk complex are largely depending on the movement task and the tested arm in chronic stroke patients with mild to moderate upper limb motor impairments. The kinematic metrics during functional movements showed different expressions and variability when compared to those of the non-functional isolated shoulder flexion, supporting the importance to assess different movement tasks in order to get a more complete picture of the patient's quality of movement. The metrics correlate at the best moderately with standard clinical tests, which underlines their benefit. Among the investigated spatiotemporal measures of shoulder–elbow coordination, curve efficiency showed promising discriminability between the affected side and the less-affected side, the factor of affected hand dominance, and upper limb functionality and correlated well with the FMMA-UE and the FMMA-UE arm subsection, respectively. Consequentially, this study contributes to novel approaches in post-stroke upper limb assessment methodologies by combining technological opportunities to measure aspects of body function during activities that are close to that of the real world and representative for the ICF activities and participation domain.

## Data Availability Statement

The raw data supporting the conclusions of this article will be made available by the authors, without undue reservation.

## Ethics Statement

The studies involving human participants were reviewed and approved by Ethikkommission Nordwest- und Zentralschweiz (EKNZ). The patients/participants provided their written informed consent to participate in this study.

## Author Contributions

AS, JV, and JH contributed to the conception and design of this study. AS organized the database. Data curation was performed by AS, JV, and JH. The methodology and statistical approach was performed by AS and reviewed by JV, JB, and AL. AS wrote the first draft of the manuscript. AL provided the resources. All the authors contributed to manuscript revision and read and approved the submitted version.

## Conflict of Interest

The authors declare that the research was conducted in the absence of any commercial or financial relationships that could be construed as a potential conflict of interest.
